# The Role of Dietary Phenolic Compounds in Epigenetic Modulation Involved in Inflammatory Processes

**DOI:** 10.3390/antiox9080691

**Published:** 2020-08-03

**Authors:** Milan Číž, Adéla Dvořáková, Veronika Skočková, Lukáš Kubala

**Affiliations:** 1 Institute of Biophysics of the Czech Academy of Sciences, 612 65 Brno, Czech Republic; dvorakova.adela@ibp.cz (A.D.); skockova@ibp.cz (V.S.); kubalal@ibp.cz (L.K.); 2 Department of Experimental Biology, Faculty of Science, Masaryk University, 625 00 Brno, Czech Republic; 3 International Clinical Research Center, St. Anne’s University Hospital, 656 91 Brno, Czech Republic

**Keywords:** diseases, immune system, inflammation, NF-κB

## Abstract

A better understanding of the interactions between dietary phenolic compounds and the epigenetics of inflammation may impact pathological conditions and their treatment. Phenolic compounds are well-known for their antioxidant, anti-inflammatory, anti-angiogenic, and anti-cancer properties, with potential benefits in the treatment of various human diseases. Emerging studies bring evidence that nutrition may play an essential role in immune system modulation also by altering gene expression. This review discusses epigenetic mechanisms such as DNA methylation, post-translational histone modification, and non-coding microRNA activity that regulate the gene expression of molecules involved in inflammatory processes. Special attention is paid to the molecular basis of NF-κB modulation by dietary phenolic compounds. The regulation of histone acetyltransferase and histone deacetylase activity, which all influence NF-κB signaling, seems to be a crucial mechanism of the epigenetic control of inflammation by phenolic compounds. Moreover, chronic inflammatory processes are reported to be closely connected to the major stages of carcinogenesis and other non-communicable diseases. Therefore, dietary phenolic compounds-targeted epigenetics is becoming an attractive approach for disease prevention and intervention.

## 1. Introduction

Nutritional phenolic compounds and their impact on human health have attracted great interest in the last three decades. Despite an increasing amount of available data, final conclusions are still diverse and further studies are necessary. Nowadays, dietary phenolic compounds are also studied from the point of view of their possible effects on epigenetic mechanisms. Although epigenetic changes occur in many diseases, only a few studies have reported the effect of natural phenolic compounds on the prevention and treatment of diseases other than cancer. There are a limited number of studies that directly describe the connection between dietary phenolic compounds, epigenetic changes, and inflammatory processes. This review discusses this issue and comments briefly on the consecutive impacts of phenolic compounds on individual diseases. For this purpose, we performed a literature search of topics, including polyphenols, epigenetics, and inflammation using the Web of Science citation indexing service. Studies describing effects of phenolic compounds other than epigenetic ones were excluded.

## 2. Dietary Phenolic Compounds

Phenolic compounds are a class of plant secondary metabolites widely present in fruits and various plant-derived beverages such as tea or wine [1], though vegetables, legumes, and grains are also non-negligible sources [2]. They generally contribute to plant defense reactions against pathogens, herbivores, or oxidative stress. Over 8000 phenolic compounds have already been described in edible plants [3]. Taken together, phenolic compounds are an important part of the human diet, as they are the most abundant antioxidants consumed by humans [4].

As mentioned above, phenolic compounds are well-known for their antioxidant properties [5,6,7]; they may protect cells against oxidative damage and, in this way, reduce the risk of various diseases associated with oxidative stress [4]. Anti-inflammatory, anti-angiogenic, and anti-cancer properties have already been described for various phenolic compounds and the positive effects of certain phenolic compounds have been observed in a wide range of non-communicable diseases (neurodegenerative diseases, chronic inflammation, cancer, cardiovascular diseases, type 2 diabetes, and obesity). Some phenolic compounds may support beneficial intestinal microflora, which could also positively impact chronic disease risk [8].

The use of phenolic compounds as a therapeutic measure can encounter certain problems. Phenolic compounds can present a potential health risk when consumed in high concentrations—for example, as food supplements instead as a natural part of the diet. Although phenolic compounds are strong antioxidants, they can also display pro-oxidative effects under certain conditions, as in the presence of transition ions. In the human body, phenolic pro-oxidants can be produced in the gastrointestinal tract when there is an abundance of phenolic compounds and, in high concentrations, could adversely affect the organism [9]. A different kind of problem is represented by isoflavones, which display estrogenic effects. It was observed that soy isoflavones may impact growth and pubertal development in children fed soy-based food in infancy. On the other hand, a potential negative effect of soy isoflavones on women with or at-risk for estrogen-sensitive breast cancer was not proven [8].

Another problem is phenolic compound bioavailability. It must be taken into consideration that while the exposure of humans to dietary phenolic compounds is frequent and long lasting, the fate of dietary phenolic compounds in the body—specifically, their bioavailability and tissue distribution—is not well understood. In particular, there is still a lack of clinical data on the effective dosage of individual phenolic compounds, the metabolic fate of phenolic compounds, their bioavailability and distribution in individual organs, tissues and cell types, the possible synergistic or antagonistic effects of individual compounds contained in various foods, and the possible interactions with gut microflora or lipid domains in cell membranes [10].

Structurally, phenolic compounds range from simple molecules to complex compounds. They are derivatives of phenylalanine or its precursor shikimic acid and are often conjugated with sugar residues, organic acids, or other phenolic molecules [2,3].

**Figure 1:**
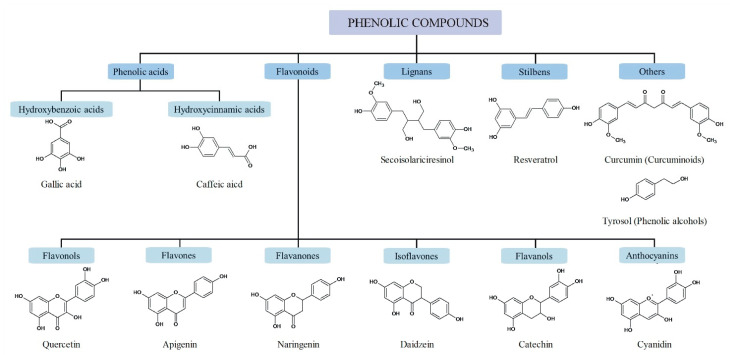
Classification and structure of the cited phenolic families.

The classification of phenolic compounds is based on their chemical structure, or, more precisely, on the number of phenol rings and the way these rings bind together [2]. Phenolic compounds are divided into at least 10 different classes, which include flavonoids, phenolic acids, lignans, stilbenes, and others [11]. The classification of selected phenolic compounds and their main sources are summarized in Table 1. The structure of the cited phenolic families is shown in Figure 1.

### 2.1. Phenolic Acids

Phenolic acids consist of derivatives of benzoic and cinnamic acids [2].

There are a few edible plants with a higher content of hydroxybenzoic acids; these include certain red fruits (blackberries, raspberries), black radish, onions, olives, and tea. Gallic acid and protocatechuic acid are the main compounds in this group; the main source of gallic acid is tea, while high levels of protocatechuic acid can be found in raspberries and olive oil [2]. Slightly less abundant is ellagic acid, which occurs mostly in berries (raspberries, strawberries), pomegranates, and nuts [31].

Hydroxycinnamic acids are a more common type of phenolic acid. The most abundant are coumaric, caffeic, ferulic, and sinapic acids. Hydroxycinnamic acids are usually found in the form of glycosylated derivatives or esters of quinic, shikimic, and tartaric acids. For example, coumaric and caffeic together with quinic acid form chlorogenic acids, which are common in various types of fruits and coffee beans [32]. Caffeic acid is, overall, the most copious phenolic acid and the most abundant hydroxycinnamic acid in fruits (especially high contents can be found in blueberries, kiwis, plums, cherries, and apples). The most abundant phenolic acid in grains is ferulic acid [2].

### 2.2. Flavonoids

Flavonoids are the largest class of polyphenols [33]. All compounds share a similar structure with two aromatic rings and are divided into subclasses based on the oxidation status of the central pyran ring (the third ring which links the two aromatic rings). Presently, six subclasses of flavonoids have been identified: flavonols, flavones, flavanones, isoflavones, anthocyanins, and flavanols [34].

#### 2.2.1. Flavonols

Flavonols are the most abundant flavonoids in food and include quercetin, kaempferol, and fisetin. Flavonols mostly occur in glycosylated form, often bound to glucose or rhamnose. High amounts of flavonols can be found in some vegetables (onion, curly kale, leeks, broccoli) and blueberries. These flavonoids are usually accumulated in the outer parts of plant as their biosynthesis is stimulated by light [2].

#### 2.2.2. Flavones

Flavones are less common than flavonols and their main representatives are luteolin and apigenin. Similarly to flavonols, they are also found in the form of glycosides. The most important sources of flavones are parsley and celery [2].

#### 2.2.3. Flavanones

High concentrations of flavanones are present only in citrus fruits; such flavanones include naringenin, hesperetin, and eriodictyol, which are found in grapefruit, oranges, and lemons, respectively. Tomatoes and mint are minor sources of flavanones. All flavanones are usually glycosylated by a disaccharide [2].

#### 2.2.4. Isoflavones

Isoflavones, also called phytoestrogens, show structural similarities to estrogens, as the configuration of their hydroxyl groups is analogous to that in the estradiol molecule. Although isoflavones are not steroids, they are able to bind to estrogen receptors, thanks to their similarity to estradiol. The most prevalent isoflavones are genistein, daidzein, and glycitein, which are often glycosylated by glucose [35]. During digestion, consumed isoflavones may be converted by colonic microflora into equol, another isoflavone, which shows even greater phytoestrogenic properties than the original isoflavones [36]. The main sources of isoflavones in the human diet are legumes, of which the most important is soya [35].

#### 2.2.5. Flavanols

Flavanols are found in both monomeric and polymeric form; the monomers are called catechins while the polymers are called proanthocyanidins [37]. Unlike other subclasses of flavonoids, flavanols do not occur in glycosylated form [2].

Catechins are the most common bioactive substances in green tea; another important source is cocoa. Red wine and some types of fruits (mostly apricots and cherries) contain slightly lower but still substantial amounts of catechins. The most abundant catechins in fruits are catechin and epicatechin, while in tea, the most common catechins are gallocatechin, epigallocatechin, and epigallocatechin gallate (EGCG) [37,38,39].

#### 2.2.6. Anthocyanins

Anthocyanins are pigments located in vacuoles of plant epidermal cells; they are usually glycosylated. The most common anthocyanin is cyanidin, with some of the highest amounts present in blackcurrants and blackberries. Anthocyanins are generally abundant in fruits, but red wine and some cereals and vegetables (eggplant, cabbage, beans, onions, radishes) are also good sources of them. Anthocyanins occur mostly in the peel and their concentration increases during fruit ripening [2].

### 2.3. Lignans

Lignans are composed of two phenylpropane units and usually occur in free form. The richest source of lignans is linseed, but smaller amounts can also be found in algae, lentils, wheat, certain vegetables (garlic, asparagus, carrots), and fruits (pears, plums). The main lignan in linseed is secoisolariciresinol; matairesinol could be found in lower concentrations. After consumption, lignans are converted to enterodiol and enterolactone by intestinal microflora [40,41,42].

### 2.4. Stilbenes

A low level of stilbenes occurs in the human diet [2], but despite this, one stilbene in particular, resveratrol, has been studied intensively because of its possible healthful effects [8]. Resveratrol is produced by plants in response to pathogen attack or stress conditions and occurs mostly in glycosylated form, named piceid [43,44]. Resveratrol and piceid are most abundant in the skins of grapes and subsequently are extracted into red wine; minor sources are peanuts and certain berries (cranberries, blueberries) [45]. Another currently studied stilbene is pterostilbene, which is the methoxylated analogue of resveratrol and occurs mainly in blueberries and grapes [46].

### 2.5. Phenolic Alcohols

The main phenolic alcohols are tyrosol and hydroxytyrosol and their richest source is virgin olive oil [47]. Tyrosol can also be found in beverages such as wine and beer [48], while hydroxytyrosol is present in red wine and is additionally generated during digestion after red wine consumption [49].

### 2.6. Curcuminoids

Curcuminoids have a symmetric molecule with two similar aromatic rings. The main curcuminoid, curcumin, can mostly be found in turmeric, which is used as a spice as well as a medicinal herb with a wide range of uses in Indian and Chinese medicine [50].

## 3. Mechanisms of Epigenetic Regulation

An epigenetic modification is defined as an alteration in gene expression that does not involve a change in the DNA sequence [51]. Epigenetic modifications determine the expression or silencing of genes. The epigenome is very stable in general, but some of these modifications can be affected by the environment (e.g., nutrition, infections, chemical agents, drugs, cytokines, or hormones) [52,53]. The epigenetic modulation of gene expression is crucial for eukaryotic life, as its dysfunction occurs in numerous human diseases [54]. These epigenetic modifications are also responsible for the regulation of the expression of genes related to the immune system; thus, they can modify innate and adaptive immune responses [52]. Epigenetic mechanisms are able to modulate host defense and determine specific immune signaling pathways. The recognition of pathogens by the innate immune system triggers the expression of genes to produce cytokines, chemokines, and transcription factors. The production of these specific inflammatory mediators can also be controlled by epigenetic mechanisms [55].

Among the most recognized epigenetic modifications are DNA methylation, histone modification, and non-coding RNA, which play an important role in cellular differentiation processes [55].

### 3.1. DNA Methylation

The methylation of DNA is an epigenetic modification in which methyl groups are added to the DNA molecule. It is a reversible covalent modification of DNA, enabled by DNA methyltransferases (DNMTs) [56]. DNMTs catalyze the addition of the methyl group from S-adenosyl-L-methionine to the C5 position of cytosines, mainly in 5’-cytosine-phosphate-guanine-3’ (CpG) islands [57]. A family of DNA methyltransferase enzymes responsible for the methylation of mammalian DNA comprises DNMT1, DNMT3A, DNMT3B, and DNMT3L. DNMT1 is the most abundant and major maintenance methyltransferase, which is responsible for the propagation of existing DNA methylation patterns. DNMT3A and DNMT3B are two related proteins that function mainly as de novo methyltransferases that set up DNA methylation patterns early in development. DNMT3L acts as a cofactor for DNMT3A during embryogenesis, but is catalytically inactive [58].

Generally, a lack of DNA methylation in promoter regions of genes is often associated with transcription activation and gene expression. Conversely, methylation in gene promoters can be responsible for gene silencing [57]. The hypermethylation of DNA CpG islands plays a significant role in the epigenetic mechanism for silencing genes crucial for cell cycle regulation, receptors, DNA repair, and apoptosis. [20]. Therefore, the regulation of DNMT activity by specific inhibitors represents a promising therapeutic target with respect to arresting abnormal cellular processes [58].

### 3.2. Histone Modification

In the eukaryotic nucleus, 147 bp of DNA associates with histone proteins to form a nucleosome—the basic structural unit of chromatin [59]. The packaging of genomic DNA in chromatin is a process intimately dependent on histone proteins that participates in the regulation of gene expression. Chromatin dynamics are facilitated by two mechanisms: complexes remodeling chromatin and enzymes modifying histone proteins [55]. Post-translational modifications of histone proteins that are essential for the epigenetic control of gene expression take place predominantly at their N-terminal tails, which project out of the nucleosomal core and are rich in lysine and arginine residues [54]. Histone modifications include methylation, acetylation, phosphorylation, citrullination (deimination), and ubiquitination, among others [56], and they work synergistically to promote transcriptional activation or repression [52,60].

#### 3.2.1. Histone Acetylation

Acetylation of lysine residues of histones is catalyzed by histone acetyltransferases (HATs) with acetyl-CoA as the acetyl group donor [61]. To balance the acetylation of histones, histone deacetylases (HDACs) remove acetyl groups from the lysine residues [55]. Histone acetylation neutralizes the positive charge of lysine residues, resulting in the weakening of histone-DNA interactions, which, in turn, leads to the decondensation of chromatin and the increased accessibility of DNA for transcription factors. Thus, it is generally accepted that acetylation is associated with transcription activation, while histone deacetylation is associated with histone condensation and transcription repression [61]. The degree of acetylation has been shown to modulate the regulation of inflammatory genes, DNA repair, and cell proliferation [55].

#### 3.2.2. Other Histone Modifications

There are other modifications of histones with significant effects on gene expression. One of these is the phosphorylation of histone proteins, which is also associated with the activation of transcription. Serine or threonine residues are accessible to phosphorylation by histone kinases, while phosphates are removed by diverse phosphatases. HMTs add methyl groups to arginine to form mono- or dimethylated residues, or to lysine, which can accept one, two, or three methyl groups [56]. The methylation of arginine can be reversed by deiminases, which block methylation of arginine by changing it into citrulline [56,61]. Further, citrullination is the conversion of arginine residues to form the nonstandard amino acid citrulline by removal of the imino group. This reaction is catalyzed by peptidyl arginine deiminases [56]. Histone arginine citrullination is often compared to histone lysine acetylation because of the loss of positive charge resulting in the decondensation of chromatin and the possible activation of transcription [54].

### 3.3. Non-Coding RNAs

Small non-coding RNAs can also affect the epigenome but these mechanisms of epigenetic control are less well understood than the mechanisms based on DNA and histone modifications [53,56]. Small non-coding RNAs are principally derived from larger RNA precursor molecules by means of cleavage with RNAse III-family enzymes and include microRNAs (miRNAs), short interfering RNAs (siRNAs), PIWI-interacting RNAs (piRNAs), and small nuclear RNAs (snRNAs) [62].

miRNAs are able to negatively control gene expression by binding target mRNA and thus by mediating its degradation and the inhibition of its translation [63]. siRNAs, which are produced by the cleavage of larger double-stranded RNA molecules (dsRNAs), can degrade homologous mRNAs and thus interfere with the translation process [64]. snRNAs control pre-messenger RNA as a part of spliceosome machinery and regulate transcription by RNA polymerase II via regulating the level of active positive transcription elongation factor b in the nucleus [65]. piRNAs associate with encoding regulatory PIWI proteins to configure a piRNA-induced silencing complex. This is connected to the post-transcriptional silencing of genes and epigenetic reprogramming [66].

In addition, miRNAs and siRNAs are both presumably involved in the process of DNA methylation and even histone modification [56,60]. Further, piRNA mediates histone H3K9 trimethylation and silences transposable elements [53].

## 4. Epigenetic Modifications in Inflammation

Inflammation, as a typical innate immune response, is very complex and relies on the precise control of many functional mechanisms acting at different levels, including also the regulation of gene expression by epigenetic modifications. While acute inflammatory response is mediated mainly by neutrophils, chronic inflammatory response is mediated mostly by monocytes and macrophages producing pro-inflammatory cytokines [67]. Inflammation is closely connected to the release of reactive oxygen species (ROS) and reactive nitrogen species (RNS) by polymorphonuclear and mononuclear phagocytic cells. Increased levels of ROS and RNS lead to the formation of oxidative stress and tissue damage [68]. Deregulation of the extent of inflammatory response is a characteristic feature of chronic inflammatory diseases. Genetic, epigenetic, and environmental factors play a role in this pathological condition [67].

### 4.1. NF-κB Signaling

Nuclear factor NF-κB is a ubiquitous transcriptional factor in eukaryotic cells and is crucial for inflammatory immune response. It modulates the regulation of many genes (for chemokines, cytokines, cell adhesion molecules, etc.) including various mediators that intensify inflammatory response and worsen the status of inflammatory disease [1,69]. NF-κB consists of different subunits, of which p65 and p50 are the most common and well-studied. In unstimulated cells, NF- κB is often inactive and forms a complex with its inhibitor subunit IκBα. NF-κB activation by different stimuli leads to degradation of the IκBα subunit, and released dimer p65-p50 is then translocated to the nucleus, where it binds to its promoters and induces the expression of inflammatory genes. The p65 subunit is the key transcriptionally active component of NF-κB [70]. Phosphorylation of the p65 subunit is required for its transcriptional regulation [71], while acetylation of the p65 subunit is necessary for its full transcriptional activity and for the regulation of NF-κB signaling. p65 acetylation and deacetylation, which are mediated by HATs and HDACs, represent an important epigenetic target during inflammation. A decreased level of p65 acetylation leads to the inhibition of NF-κB target gene expression, such as the expression of interleukin-6 (IL-6) or TNF-α (tumor necrosis factor alpha) genes, and can even prevent the translocation of p65 to the nucleus. The regulation of NF-κB also affects the expression of COX (cyclooxygenase) and iNOS (inducible nitric oxide synthase). The modulation of p65 acetylation thus represents a promising target in chronic inflammation therapy [1,70]. Additionally, two closely related HATs, p300 and CBP (CREB-binding protein), which are transcriptional cofactors, exhibit a strong synergism with the p65 subunit [12].

The generation of ROS also leads to the activation of various signaling pathways such as extracellular signal-regulated kinases (ERKs). It can further induce NF-κB-dependent immune response and alterations in histone protein acetylation/deacetylation status, which finally results in changed transcription initiation [68]. All these ROS-mediated mechanisms result in augmented pro-inflammatory cytokine levels, which contribute to the chronicity of the inflammatory process.

### 4.2. Chronic Inflammation

During chronic inflammatory processes, the epigenetic mechanisms involved in the regulation of gene expression are modified. For example, ROS and RNS generated during oxidative stress may cause a loss of DNA methylation after mitosis, since DNMT1 does not recognize oxidatively modified methyl groups. On the other hand, halogen derivatives produced by inflammatory processes imitate cytosine methylation, resulting in increased methylation by DNMT1. The levels of some pro-inflammatory cytokines (e.g., TNF-α) are tightly modulated by epigenetic mechanisms. In contrast, some other cytokines (e.g., IL-6) can provoke histone protein modifications and DNA methylation and thus regulate other gene expressions and processes. Chronic inflammatory processes are generally characterized by epigenetic changes based on the enrichment of hypo-acetylated histones and hypermethylated CpGs. The modulation of both HDACs and DNMT1 activities is thus therapeutically beneficial in chronic inflammatory diseases and both enzymes represent a transcriptional repression complex [67].

## 5. Epigenetics, Inflammation, and Phenolic Compounds

Phenolic compounds have the ability to modulate gene expression through the regulation of epigenetic mechanisms including either DNA methylation, histone modification, or miRNA expression. In general, several phenolic compounds are able to activate HDACs (e.g., fisetin), activate (e.g., genistein) or inhibit (e.g., EGCG, curcumin) HATs, activate (e.g., resveratrol) sirtuins (SIRT), and inhibit DNMTs (e.g., EGCG) [14,21,23,27,72]. Some phenolic compounds are associated with the regulation of miRNA expression (e.g., genistein, curcumin) [17,28]. Many phenolic compounds exhibit anti-inflammatory properties and have been shown to inhibit the secretion and production of pro-inflammatory cytokines and decrease the production of ROS and nitric oxide (NO) [73].

On the basis of available data, it is generally accepted that phenolic compounds are also able to modify epigenetic mechanisms involved in the modulation of the immune system and diseases connected to inflammation [72]. Many phenolic compounds show the potential to modulate NF-κB activity and chromatin remodeling through the regulation of HDACs and HATs [1]. The epigenetic effects of selected phenolic compounds related to inflammation are summarized in Table 1. Animal and human studies in which the effects of phenolic compounds on epigenetic changes under inflammatory conditions were studied are listed in Table 2 and Table 3.

### 5.1. Phenolic Acids

Gallic and ellagic acids can affect the activity of HATs and HDACs in monocytic cells with induced inflammation. Kiss and co-workers proved that both acids decreased HAT activity in TNF-α-activated human monocytic (THP-1) cells and that ellagic acid also increased HDAC activity. This led to the attenuation of inflammatory response and improved the survival of cells [12].

Another phenolic acid, dihydrocaffeic acid (DHCA), which is a metabolite of caffeic acid, influences the methylation level of DNA. It is known that stressful conditions can change the methylation level of DNA in humans and animals, which also affects immunity, as the methylation of genes related to immunity leads to less effective immune response [78,79]. Blaze and colleagues proved that treatment with DHCA caused a decrease in DNA methylation level in peripheral leucocytes from mice exposed to stressful conditions [13]. In another experiment, DHCA also decreased the level of DNA methylation in human and mice peripheral leucocytes exposed to lipopolysaccharide (LPS) in vitro, which means DHCA can exhibit an anti-inflammatory effect [13].

### 5.2. Stilbenes

Resveratrol exhibits anti-cancer, anti-oxidative, and anti-angiogenic properties with potential benefits for human immunity or in the treatment of some diseases including diabetes, rheumatoid arthritis, metabolic disorder, and cardiovascular or neurodegenerative diseases. It has been shown that resveratrol directly targets immune cells, such as macrophages, large lymphocytes, and dendritic cells [58,80,81,82]. It can also affect several epigenetic targets including DNMTs and HDACs, especially SIRT1 deacetylase from class III HDACs [23,58,82,83,84]. SIRT1 can deacetylate the p65 subunit of NF-κB and also inhibit NF-κB-mediated inflammation [85]. Schug and co-workers demonstrated that SIRT1 inhibited the transcriptional activity of NF-κB through the deacetylation of the p65 subunit, affecting the expression of inflammatory cytokines by macrophages [86]. TNF-α induced NF-κB p65 expression in human umbilical vein endothelial cells (HUVECs) was diminished by SIRT1 activation triggered by resveratrol treatment [24]. In another experiment, Bo and colleagues found that SIRT1 expression in peripheral blood mononuclear cells was increased in the case of type 2 diabetes mellitus patients receiving resveratrol for six months. The elevation of SIRT1 led to significantly reduced levels of H3K56 acetylation and it is known from previous research that a high acetylation level of H3K56 is related to genes playing an important role in the long-term effect of hyperglycemia on the body [76]. Besides SIRT1, resveratrol can also affect the methylation of DNA. Lou et al. observed that longer resveratrol administration to diabetic rats led to a decline in the levels of pro-inflammatory cytokines (IL-1, IL-6, TNF-α, and interferon-γ) and enhanced the production of anti-inflammatory IL-10 in the cells of arterial intima. At the same time, they detected a higher level of the methylation of CpG islands in these pro-inflammatory genes and a lower level of CpG methylation in the IL-10 gene compared to untreated diabetic individuals. These results indicate that resveratrol can decrease inflammation also through changes in DNA methylation and has a protective effect on the aorta or other arteries under hyperglycemic conditions [25]. The available experimental data also suggest that resveratrol and its analogs are able to modulate miRNA expression in various diseases [26,87].

### 5.3. Flavonols

Quercetin has been shown to inhibit the production of COX and lipoxygenase, inhibit the molecular level of NF-κB, and block pro-inflammatory cytokine production or inhibit mitogen-activated protein kinase; it thus possesses anti-inflammatory, anti-cancer, and anti-diabetic qualities [73,80]. Quercetin was reported to increase histone H3 acetylation in HL-60 (human promyelocytic leukemia cells); therefore, quercetin shows potential for the activation of HATs or the inhibition of HDACs [88] and probably possesses histone demethylase inhibitor activity [89]. On the other hand, Xiao et al. found that quercetin suppressed p300 activity in human breast cancer and endothelial cells and thus reduced the p300-mediated acetylation of NF-κB [15]. Ruiz and colleagues demonstrated that quercetin inhibited NF-κB binding to the pro-inflammatory interferon-γ-inducible protein (IP-10) and macrophage inflammatory protein 2 (MIP-2) gene promoters in murine intestinal epithelial cells, therefore further blocking cofactor recruitment and HATs activity at the chromatin of these promoters. In addition to reducing HAT activity, quercetin inhibited the TNF-induced acetylation and phosphorylation of histone H3 and NF-κB cofactor CBP/p300 at the IP-10 and MIP-2 gene promoter [90].

Fisetin also exhibits anti-inflammatory properties and may provide potential benefits in the treatment of several diseases. For example, diabetes is closely associated with chronic inflammation, as high glucose levels have been implicated in the activation of histone acetylation and the activation of NF-κB. Kim et al. observed that fisetin suppressed pro-inflammatory cytokine release via the NF-κB signaling pathway in THP-1 cells. The supplementation of cells with fisetin activated HDACs and suppressed HATs, resulting in deacetylation of the p65 subunit of NF-κB and finally in diminished pro-inflammatory cytokine release [14].

### 5.4. Flavones

Luteolin is reported to have antioxidant, anti-inflammatory, and anti-cancer properties. It is related to the activation of nuclear factor 2 (erythroid-derived 2)-like 2 (Nrf-2), a transcription factor which causes the transactivation of antioxidant genes [91,92]. Luteolin also displays similar effects as fisetin, as it activates HDACs and inhibits HATs in high glucose-treated THP-1 cells. Luteolin inhibits HAT activity and the expression of CBP/p300 protein, which results in deacetylation of the p65 subunit and subsequently in the decreased activity of NF-κB. This leads to the suppression of inflammatory cytokine release, such as IL-6 and TNF-α [16]. Recently, Kim et al. found that luteolin in combination with fisetin not only suppressed HAT and NF-κB activity and inflammatory cytokine release in THP-1 cells, but also significantly inhibited ROS production and activated SIRT1 expression [93].

### 5.5. Flavanols

The tea polyphenol EGCG exhibits anti-NF-κB activity in several pathological conditions, such as chronic inflammation or cancer. Choi and colleagues proved that EGCG was responsible for HAT inhibition and induced hypoacetylation of the p65 subunit. Furthermore, EGCG reduced the TNF-α-induced expression of IL-6, COX-2 and iNOS in HEK293, THP-1 cells, and even primary peritoneal macrophages [21]. EGCG was also shown to inhibit DNMTs and thus reactivate methylation-silenced genes in cancer cell lines [20]. Recently, it was demonstrated that EGCG mitigates vascular inflammatory response through a repressive epigenetic effect on the NF-κB signaling pathway [94]. The positive effect of EGCG was also observed in the case of regulatory T cells (Tregs), which are negative regulators of inflammation. Tregs function and their number are often decreased in obese individuals compared with lean subjects. The role of EGCG in the modulation of Tregs was studied in obese and lean human subjects in vitro; Tregs were isolated and cultured in the absence or presence of EGCG. It was observed that EGCG treatment enhanced HDAC activity and decreased NF-κB activity in both groups. Furthermore, EGCG also increased the production of anti-inflammatory cytokine IL-10 by suppressing the NF-κB signaling pathway [95].

Cordero-Herrera and colleagues demonstrated that epicatechin, a main cocoa flavanol, prevented the increased acetylation of H3K9 and the dimethylation of H3K4, and also decreased the dimethylation of H3K9 in THP-1 cells cultured under high glucose conditions by affecting HDAC4 levels and HAT activity. They further showed that both the expression level of NF-κB and the release of TNF-α were decreased in these cells [22]. Crescenti and co-workers showed that the consumption of cocoa led to a decreased DNA methylation level in human peripheral mononuclear leucocytes [77]. It was confirmed previously that elevated DNA methylation in these cells is associated with various non-communicable diseases such as cardiovascular disorders and obesity [96] or insulin resistance [97].

### 5.6. Isoflavones

Genistein has been shown to possess anti-diabetic, anti-inflammatory, and anticancer properties. It is able to affect cancer cell survival and activate tumor suppressor genes by epigenetic changes [18,98]. Various cancers have been associated with the hypermethylation of CpG islands at the regulatory sites of tumor suppressor genes with subsequent gene silencing and genistein has been shown to decrease DNMT activity. Furthermore, genistein is able to increase HAT activity, as histone acetylation is related to a loosened chromatin structure and the induction of tumor suppressor gene expression in various cancer cell types [19,98]. In another study, genistein was able to increase SIRT1 levels in a model of ovariectomized diabetic rats, which led to a subsequent decrease in NF-κB and IL-1β protein levels, this providing evidence of the anti-inflammatory effect of genistein [18]. Recently, Zhang et al. demonstrated that genistein played an important role in the prevention of atherosclerosis through the regulation of miRNA expression. This study indicates that genistein could reverse oxidized low-density lipoprotein-induced inflammation through the regulation of miRNA-155/SOCS1 (suppressor of the cytokine signaling-1), which was accompanied by the inhibition of the NF-κB signaling pathway in HUVECs [84].

### 5.7. Curcuminoids

Curcumin is well-known for its anti-inflammatory, anti-oxidative, and anti-lipidemic properties and is able to modulate several diseases (e.g., neurological disorders, diabetes) via epigenetic regulation. It suppresses the function of NF-κB by decreasing the acetylation level of the p65 subunit [70], which regulates the anti-inflammatory response of the enzymatic activities of COX and iNOS. Curcumin also downregulates the expression of NF-κB-related gene products (e.g., TNF-α, IL-1, IL-6, IL-8, adhesion molecules) [74,99] and modulates signaling pathways controlling anti-oxidative properties through the regulation of Nrf-2 [29]. Several studies have recently shown that curcumin can affect DNMTs and HDACs and thus reverse the silencing of key genes [29,30,75]. Moreover, curcumin has also been shown to alter the profiles of miRNA expression [28] and to inhibit HATs [27]. HAT inhibition resulted in the significantly reduced acetylation of histone H3 levels in the IL-6 promoter, as well as decreased IL-6 mRNA expression and IL-6 protein release by rheumatoid arthritis synovial fibroblasts [100]. In addition, in a study using THP-1 cells as a model for human monocytes, curcumin was reported to have an effect on histone acetylation and pro-inflammatory cytokine secretion under high-glucose conditions. The results showed that curcumin treatment not only significantly reduced HAT activity and the level of p300 (a co-activator of NF-κB), but also induced HDAC2 expression. The results indicate that curcumin decreases high glucose-induced cytokine production in monocytes via epigenetic changes involving NF-κB [70]. A similar effect of curcumin (increased HDAC and reduced HAT activity) was also observed in TNF-α-activated THP-1 cells [12]. In another study, Yuan and colleagues investigated the role of curcumin-mediated epigenetic modulation of the expression of TREM-1 (triggering receptor expressed on myeloid cells 1) proteins. TREMs constitute a family of immunoglobulin cell surface receptors expressed on macrophages and neutrophils, which are capable of regulating various immunological events in both innate and adaptive immune cells. Yuan and colleagues demonstrated that curcumin inhibited the methylation and acetylation of H3K4 and p300 in the TREM-1 promoter in vitro in bone marrow derived macrophages and in vivo in a septic lung injury model, which led to the reduced binding of p65 to the TREM-1 promoter in response to LPS. Their study also indicated that the inhibition of TREM-1 by curcumin is independent of ROS production [19]. Curcumin also inhibits the phosphorylation and degradation of IκBα and the subsequent translocation of the p65 subunit of NF-κB to the nucleus [101].

## 6. Conclusions

Many phenolic compounds exhibit anti-inflammatory properties and have been shown to inhibit the secretion and production of pro-inflammatory cytokines and decrease the production of reactive oxygen and nitrogen species. Recent findings suggest that phenolic compounds can regulate epigenetic mechanisms involved in immune system modulation. Phenolic compounds have the ability to modulate gene expression through the regulation of epigenetic mechanisms including DNA methylation, histone modification, and miRNA expression.

**Figure 2:**
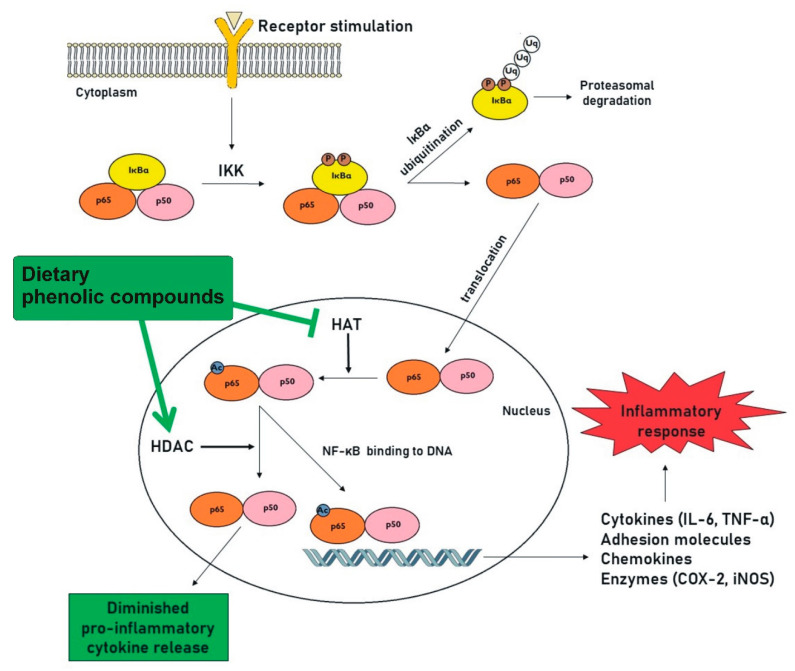
The regulation of inflammatory response by dietary phenolic compounds through HAT inhibition or HDAC activation. NF-κB is a nuclear factor responsible for the transcription of genes connected to inflammatory immune response. It is found in cytoplasm associated with its inhibitory subunit IκBα, which masks the nuclear translocation signal. Upon cell stimulation, the inhibitory subunit is phosphorylated by IκB kinase (IKK), which leads to its ubiquitination and degradation by proteasome. The released NF-κB dimer (consisting of the p65 and p50 subunits) is then translocated into the nucleus and initiates the expression of genes, including genes for various mediators that intensify inflammatory response. HATs and HDACs have been implicated in regulating NF-κB activity. While HATs have a role in NF-κB-mediated transcriptional activation, HDACs have been shown to reverse this process and to repress NF-κB-mediated gene transcription. Many dietary phenolic compounds show a potential to modulate NF-κB-mediated inflammation through the regulation of HAT or HDAC activity. Ac: acetylation; COX-2: cyclooxygenase-2; HAT: histone acetyltransferase; HDAC: histone deacetylase; IKK: IκB kinase; IL-6: interleukin 6; iNOS: inducible nitric oxide synthase; NF-κB: nuclear factor kappa B; P: phosphorylation; TNF-α: tumor necrosis factor alpha; Uq: ubiquitination.

In addition, however, the regulation of HATs, HDACs, and SIRT1 activity, which all influence NF-κB signaling, seems to be a crucial mechanism of the epigenetic control of inflammation by phenolic compounds (Figure 2). Furthermore, this mechanism occurs to a greater or lesser extent in many different phenolic classes. The modulation of epigenetic modifications by phenolic compounds is, therefore, of great interest in the context of clinical approaches to immune-mediated diseases and diseases such as rheumatoid arthritis, cancer, cardiovascular disease, atherosclerosis, metabolic disorders, neurodegenerative diseases, obesity, and diabetes.

Taking all the above information together, the use of dietary phenolic compounds to induce epigenetic modifications appears to be a promising approach with respect to disease prevention and the development of treatment strategies. Nevertheless, the role of phenolic compounds in the regulation of epigenetic mechanisms is complex and still poorly understood. Thus, it represents an attractive field of research.

## Figures and Tables

**Table 1:** Phenolic compounds and their epigenetic effects.

Classification		Examples of Phenolic Compounds	Main Sources	Possible Targets	References
**Phenolic Acids**	Hydroxybenzoic acids	**gallic**, protocatechuic, **ellagic acids**	berries, olive oil, tea	HATs, HDACs	[12]
	Hydroxycinnamic acids	coumaric, ferulic, sinapic, **caffeic acids**	fruits (blueberries, cherries, apples), grains	DNMTs	[13]
Flavonoids	Flavonols	**quercetin**, kaempferol, **fisetin**	vegetables (leek, broccoli), blueberries	HATs, HDACs	[14,15]
	Flavones	**luteolin**, apigenin	parsley, celery	HATs, HDACs	[16]
	Flavanones	naringenin, hesperetin, eriodictyol	citrus fruits	not determined	
	Isoflavones	**genistein**, daidzein, glycitein	legumes (soya)	DNMTs, HATs, SIRT1, miRNA	[17,18,19]
	Flavanols	catechin, **epicatechin**, gallocatechin, epigallocatechin, **epigallocatechin-3-gallate**	green tea, cocoa	DNMTs, HATs. HDACs, HMTs	[20,21,22]
	Anthocyanins	cyanidin	fruits (blackcurrants, blackberries)	not determined	
Lignans		secoisolariciresinol, matairesinol	linseed	not determined	
Stilbenes		**resveratrol**, pterostilbene	grapes, red wine, blueberries	SIRT1, DNMTs, miRNA	[23,24,25,26]
Phenolic alcohols		tyrosol, hydroxytyrosol	virgin olive oil	not determined	
Curcuminoids		**curcumin**	turmeric	DNMTs, miRNA, HATs, HDACs	[27,28,29,30]

The table summarizes classification of phenolic compounds, their main sources, and possible epigenetic targets in inflammation. The bold phenolic compounds are discussed in the text in more details. DNMTs: DNA methyltransferases; HATs: histone acetyltransferases; HDACs: histone deacetylases; HMTs: histone methyltransferases; miRNA: microRNA; SIRT: sirtuin.

**Table 2:** Summary of the studies performed on animal models.

Species	Strain Characteristics	Sample Size (per Group)	Pathological Condition	Type of Study	Phenolic Compound Intervention	Reference
Mus musculus	CD45.2 + C57BL/6	*n* = 9–10	repeated social defeat stress	observational, cross-sectional	dihydrocaffeic acid 5 mg/kg/d for 2 weeks, delivered through drinking water, treatment repeated 4 months later	[13]
Mus musculus	BALB/c 6 weeks old 20–25 g body weight	*n* = 3		intervention	epigallocatechin-3-gallate 100 mmol/L, 50 mmol/L	[21]
Mus musculus	C57BL/6J males 6–8 weeks old 20–30 g body weight	*n* = 12	inflammation	intervention	curcumin 200 mg/kg (i.p.)	[74]
Rattus norvegicus	Sprague–Dawley females 250–300 g body weight	*n* = 6	inflammation	intervention	curcumin 40 mg/kg (final injection volume of 0.2 mL)	[75]
Rattus norvegicus	Wistar females 10 weeks old 180–220 g body weight	*n* = 10	ovariectomized diabetic rat	intervention	genistein 1 mg/kg/day, subcutaneously, for 8 weeks	[18]
Rattus norvegicus	Sprague–Dawley males two weeks old	*n* = 19 (normal group), 16 (normal intervention group), 29 (diabetic group), 25 (diabetic intervention group)	diabetes	intervention	resveratrol intraperitoneally 10 mg/kg/day, for 10 weeks	[25]

**Table 3:** Summary of human studies.

Type of Study	Sample Size	Participant Characteristics	Dietary Intervention	Disease	Reference
intervention	*n* = 128	age ≥40 years	one capsule/day of resveratrol 500 mg/day (*n* = 43), 40 mg/day (*n* = 43) or placebo (*n* = 42), for 6 months	type 2 diabetes mellitus	[76]
intervention	*n* = 214	men and women 20 years of age	Treated group (45 men and 65 women) received the cocoa cream product (13 g/unit; 1 g cocoa/unit, 6 units/d; 6 g cocoa/d) for 2 weeks. Control group consisted of 46 men and 58 women.	cardiovascular disease risk	[77]

## List of Abbreviations

CBP	CREB-binding protein
CpG	5’-cytosine-phosphate-guanine-3’
COX	cyclooxygenase
DHCA	dihydrocaffeic acid
DNMT	DNA methyltransferase
EGCG	epigallocatechin gallate
ERK	extracellular signal-regulated kinase
HAT	histone acetyltransferase
HDAC	histone deacetylase
HMT	histone methyltransferases
HUVEC	umbilical vein endothelial cells
IKK	IκB kinase
IL	interleukin
iNOS	inducible nitric oxide synthase
IP-10	interferon-γ-inducible protein
LPS	lipopolysaccharide
MIP	macrophage inflammatory protein
miRNA	microRNA
NF-κB	nuclear factor kappa B
Nrf-2	nuclear factor 2 (erythroid-derived 2)-like 2
piRNA	PIWI-interacting RNA
RNS	reactive nitrogen species
ROS	reactive oxygen species
siRNA	short interfering RNA
SIRT	sirtuin
snRNA	small nuclear RNA
SOCS	suppressor of the cytokine signaling
TNF-α	tumor necrosis factor alpha
Tregs	regulatory T cells
TREM-1	triggering receptor expressed on myeloid cells 1
